# Partial microduplication in the histone acetyltransferase complex member *KANSL1* is associated with congenital heart defects in 22q11.2 microdeletion syndrome patients

**DOI:** 10.1038/s41598-017-01896-w

**Published:** 2017-05-11

**Authors:** Luis E. León, Felipe Benavides, Karena Espinoza, Cecilia Vial, Patricia Alvarez, Mirta Palomares, Guillermo Lay-Son, Macarena Miranda, Gabriela M. Repetto

**Affiliations:** 1 Centro de Genética y Genómica, Facultad de Medicina, Clínica Alemana Universidad del Desarrollo, Santiago, Chile; 2 Hospital Dr. Roberto del Río, Santiago, Chile; 3 Fundación Gantz, Santiago, Chile; 4 Hospital Dr. Luis Calvo Mackenna, Santiago, Chile; 5 Hospital Padre Hurtado, Santiago, Chile

## Abstract

22q11.2 microdeletion syndrome (22q11.2DS) is the most common microdeletion disorder in humans, with an incidence of 1/4000 live births. It is caused by a heterozygous deletion of 1.5–3 Mb on chromosome region 22q11.2. Patients with the deletion present features that include neuropsychiatric problems, craniofacial abnormalities and cardiovascular malformations. However, the phenotype is highly variable and the factors related to the clinical heterogeneity are not fully understood. About 65% of patients with 22q11.2DS have congenital heart defects (CHD). The main goal of this study was to identify common CNVs in 22q11.2DS patients that could be associated with the incomplete penetrance of CHD. Analysis of genomic DNA from 253 patients with 22q11.2DS using array technology showed an association between a microduplication located in region 17q21.31 and CHD (p-value = 0.023, OR = 2.75, 95% CI = 1.17–7.03). This region includes the first three exons of *KANSL1* gene. Bioinformatic analysis showed that *KANSL1* and *CRKL*, a gene in the commonly deleted region of 22q11.2DS, are part of the same regulatory module in a miRNA-mRNA network. These results show that a *KANSL1* microduplication, in combination with the 22q11.2 deletion, is associated with increased risk of CHD in these patients, suggesting that *KANSL1* plays a role as a modifier gene in 22q11.2DS patients.

## Introduction

22q11.2 deletion syndrome (22q11.2DS, OMIM #192430, #188400) is the most common microdeletion syndrome in humans and is associated with a wide variety of phenotypic features, including congenital heart defects (CHD), craniofacial anomalies, intellectual disability, increased risk of psychosis and schizophrenia, among others^1, 2^. Ninety percent of the patients have a ~3 Mb hemizygous deletion and less than 10% of patients have a smaller 1.5 or 2 Mb deletion^3, 4^. Interestingly, different deletion sizes have no apparent effects in disease severity^5^. In addition, the phenotype is highly variable even within the patients who share the same 3 Mb deletion. This heterogeneity may involve a genetic component such as single nucleotide variants (SNVs) or copy number variations (CNVs), which could be affecting modifier genes^6^. CNVs are known contributors to complex diseases including CHD^7^, being potential modifiers for several different diseases^8–10^.

Little is known about genetic modifiers that may influence the clinical variability observed in patients with 22q11.2DS. Putative modifier genes have been identified in mouse models and include *FGF8*, *FGF10*, *GBX2*, *PITX2*, *CRKL*, *VEGF*, *TGFβ*, *CHRD* and *SHH* (reviewed in Aggarwal *et al*.)^6^, but their role in humans with 22q11.2DS has not been established. To date, only three studies have performed genome-wide search strategies for modifiers of CHD in 22q11.2DS^11–13^. In these studies, 22q11.2DS patients with and without structural heart defects were compared.

In the first study, Mlynarski *et al*. performed a genome-wide association study using microarray technology. The study used probe-intensity information from microarray plus bioinformatics processing to identify CNVs, finding a statistically significant association between a duplication encompassing the *SLC2A3* gene and CHD phenotype in 22q11.2DS patients (p-value = 2.68 × 10^−4^, OR = 5.08 [95% confidence interval 2.0–17.51]). The authors show that the *SLC2A3* duplication by itself is not pathogenic unless it is inherited in combination with 22q11.2 deletion^11^. Moreover, functional studies in mice show that *SLC2A3* gene is a genetic modifier of the cardiac phenotype. The *SLC2A3* gene, also known as *GLUT3*, encodes a glucose transporter, which is important in tissues with high energy demands and metabolic rates, as cardiac myocytes and brain, among others.

The second study performed whole exome sequencing in 184 patients with 22q11.2DS, finding enrichment for rare deleterious SNVs in *MINA*, *RREB1, KDM7A* and *JMJDC1* genes in the CHD group compared to the non-CHD group. These genes code for proteins related to epigenetic regulation that activates transcription by demethylation of histones, mainly H3K9 and H3K27. The results of this study suggest that variants in histone modification genes increase the risk of CHD in the presence of deletion^13^.

The third study analyzed rare CNVs using Affymetrix SNP 6.0 technology. The authors demonstrated no significant difference in overall burden of rare CNVs in patients with CHD compared to controls. However, an enrichment of CNVs overlapping with protein-coding cardiac-related genes was found in 22q11.2DS individuals with heart defects (n = 607) compared to normal hearts (n = 339). Furthermore, network analysis revealed that CNVs in specific cardiac networks, such as Wnt signaling, were overrepresented in 22q11.2DS CHD cases but not in 22q11DS, controls suggesting that specific CNVs located outside of the 22q11.2 region may increase the risk for CHDs in some 22q11DS patients^12^.

Although these genes were identified as genetic modifiers of CHD phenotype, they explain only a small proportion of the incomplete penetrance of CHDs in 22q11.2DS patients, suggesting that other modifiers of this condition exist. To search for new genetic modifiers and to address the effect of CNVs in a Chilean cohort of patients with 22q11.2DS, a genome-wide search was performed using Affymetrix SNP 6.0 arrays (Santa Clara, CA). First, the overall burden of CNVs in 22q11.2DS patients with and without CHD was compared, including absolute CNV counts and genomic length, and then proceeded to perform association analysis between CHD phenotype and CNVs. To minimize false positives, a combination of PennCNV for CNV detection and ParseCNV for association study, followed by a validation of the CNVs with a second independent technique was chosen^14, 15^. Results indicated that a microduplication in the first three exons of KAT8 Regulatory NSL Complex Subunit 1 gene (*KANSL1*) is associated with CHD. Finally, a network integrative analysis approach was performed with computational biology tools to identify a biological interaction between *KANSL1* gene and genes within 22q11.2 deletion.

## Results

A genome-wide analysis using Affymetrix SNP 6.0 arrays in cohort one was performed, corresponding to 253 patients with 22q11.2DS. After stringent filter criteria detailed in Methods, 39 samples were excluded from the analysis (28 with CHD, 11 without): 29 for Log R Ratio (LRR) standard deviation greater than 0.45, four for quality control in wave adjustment and six for having over 100 CNV counts. In summary, 214 samples from unrelated subjects with 22q11.2DS (105 CHD and 109 without CHD) passed quality controls. The association between 22q11.2 deletion size and CHD phenotype was first assessed. There were 195 patients (91%) with a 3 Mb deletion (97 CHD and 98 No-CHD), 6 patients (2.8%) with 2 Mb (3 CHD and 3 No-CHD), and 13 (6.1%) with 1.5 Mb deletion (5 CHD and 8 No-CHD) (χ^2^ = 0.63, p-value = 0.73), showing no association between deletion size and CHD phenotype, consistent with previous publications^11, 16^.

### CNV analysis

**Figure 1:**
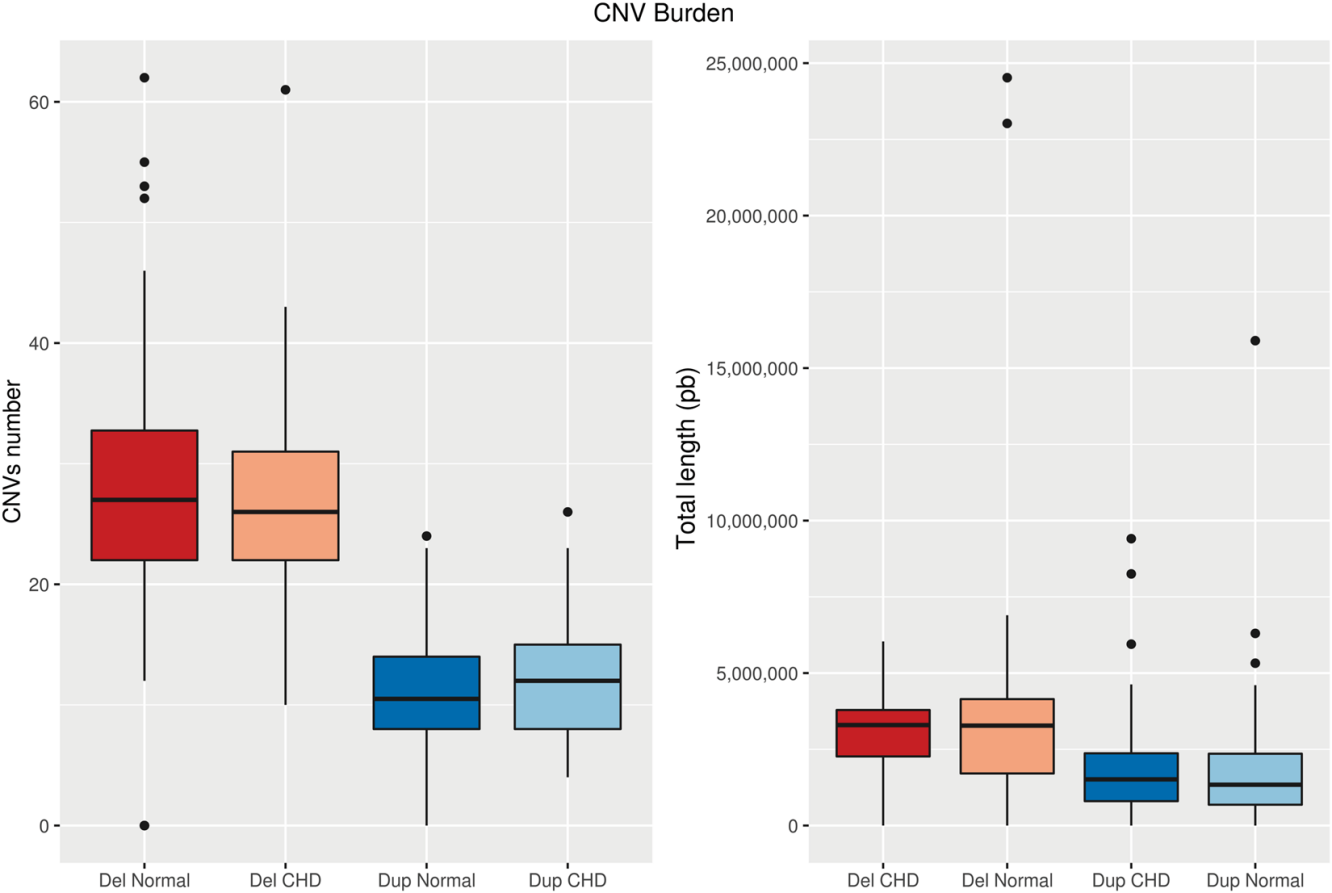
Total CNV burden in 22q11.2DS patients. Standard box-and-whiskers plot for the distribution of the total length and number of CNV (deletions and duplications) in CHD vs. no- CHD. Boxes represent the 1st and 3rd quartiles of each distribution, thick horizontal lines represent the median value, and circles represent outliers.

There were no statistically significant differences in global CNV burden between CHD and no-CHD groups. Global CNV burden measures included average CNV length (2.7 Mb for CHD group vs. 2.6 Mb for no-CHD group) and average total number of CNVs per individual (18 for CHD group vs. 19 for no-CHD group). The same analysis was performed comparing only deletions between CHD and no-CHD groups and then comparing only duplications. No statistically significant differences were observed in this analysis (Fig. 1).

Then, the statistical association of CNVs with the cardiac condition was assessed by using the overlapping regions method with the ParseCNV pipeline^15^. None of the CNVs reached the uncorrected p-value that met conservative multiple-testing significance of association (p < 5 × 10^−4^)^15^. Therefore, the top-ranked CNVs obtained by this method with an uncorrected cutoff of p < 0.05 were selected, in search of biologically plausible associations. Eleven losses and three gains were found with differences in frequencies between CHD and no-CHD groups, ranging in size from 0.122 to 117.4 kb. These CNVs are listed in Table 1 by ascending p-value.

**Table 1:** List of top-ranked CNVs associated with CHD in 22q11.2DS patients.

CNV (hg19)	CNV size (bp)	Two tailed p value	OR (95% CI)	gain/loss	CNV Cases (n = 105)	CNV Controls (n = 109)	Distance to closest gene (bp)	Putative gene/s affected by CNV
chr13:84161129–84161251	122	0.013	14.3 (0.80–257.22)	loss	6	0	290092	*SLITRK1*
chr19:43646784–43691318	44534	0.013	14.30 (0.80–257.22)	loss	6	0	0	*PSG5*
chr16:55796389–55796594	205	0.014	2.92 (1.26–7.45)	loss	20	8	0	*CES*
chr1:16869363–16986851	117488	0.017	5.28 (1.32–38.34)	gain	10	2	0	*DQ585677*, FLJ00313.MST*1*, *NBPF1*, *NBPF10*
chr6:32570046–32574381	4335	0.017	7.88 (1.38–200.89)	loss	8	1	12484	*HLA-DRB1*, *HLA-DRB5*
chr3:148963395–148968121	4726	0.017	5.28 (1.32–38.34)	loss	10	2	23563	*CP*
chr17:44176984–44272000	95016	0.023	2.75 (1.17–7.03)	gain	19	8	0	*KANSL1*
chr15:22548025–22571758	23733	0.024	3.02 (1.18–8.88)	loss	16	6	74672	*IGH*
chr17:1619932–1680318	60386	0.028	11.99 (0.65–219.50)	loss	5	0	0	*SERPINF1*, *SERPINF2*, *WDR81*
chr8:7826922–7866162	39204	0.028	11.99 (0.65–219.50)	loss	5	0	0	*DEFB109*
chr11:5789601–5794708	5107	0.035	2.22 (1.09–4.66)	loss	26	14	0	*TRIM22*, *TRIM5*
chr13:57758270–57778380	20110	0.037	2.34 (1.09–5.31)	loss	22	11	13918	*PRR20*
chr3:89394592–89417171	22579	0.038	3.29 (1.09–12.48)	loss	12	4	0	*EPHA3*
chr11:18959245–18960115	870	0.042	2.42 (1.06–5.95)	gain	19	9	2696	*MRGPRX1*

A summary of top-ranked CNVs associated with CHD is shown.

### Gene prioritization

To prioritize a candidate CNV region, a network analysis of genes located in the top-ranked 11 deletions and three duplications with phenotype information was performed using the PhenogramViz^17^ app available as a plugin for CytoScape^18^. To establish a relationship with the phenotype of interest, we used Human Phenotype Ontology (HPO) terms associated with structural heart defects.

**Figure 2:**
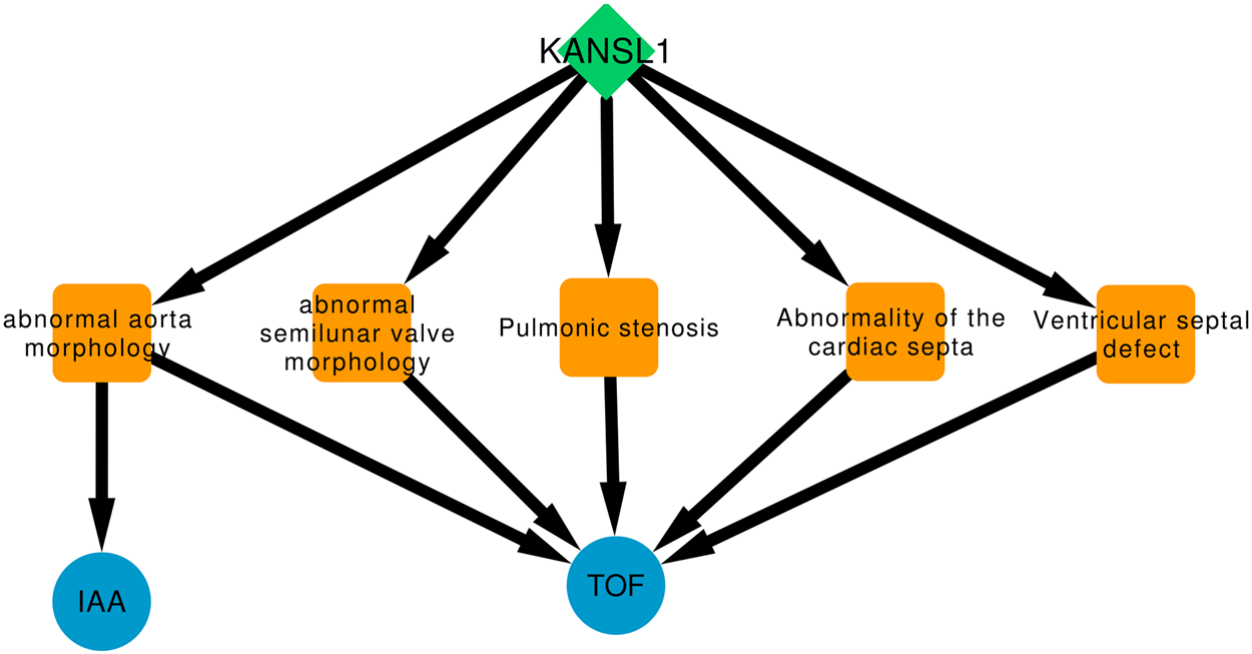
Phenogram of associated CNVs. Human genes located or overlapped with identified CNVs are showed as green diamonds, phenotypes found in the 22q11.2DS patients are displayed as blue circles and the common ancestors (link between gene’s phenotype annotation and patient’s phenotypes) are displayed as orange squares. IAA = Interrupted Aortic Arch; TOF = Tetralogy of Fallot.

Briefly, PhenogramViz integrates data from different sources (including OMIM, MGI, ZFIN and Orphanet) with the selected HPO terms and then ranks CNVs according to pathogenicity. For this study, the CNV located in chr17:44176984-44272000 ranked as number one because it was the only pathogenic CNV in association with CHD-related HPO terms. This genomic region partially overlaps with the first three exons of *KANSL1*. Observations indicate that *KANSL1* is highly interconnected with the selected HPO terms in the phenogram created with genes overlapping CNV associated regions (Fig. 2).

### Frequency of *KANSL1* duplication

The frequency of *KANSL1* microduplication was calculated in three Chilean cohorts. The first is the 22q11.2DS cohort; the frequency of CHD was 49% (n = 105), and the frequency of *KANSL1* duplication was 12.61% (n = 27). In patients with the partial *KANSL1* microduplication (n = 27, 19 patients with CHD and 8 without CHD), CHD frequency was 70%, showing an enrichment of CHD in these subjects. This increase in frequency suggests an association between *KANSL1* microduplication and CHD or that KANSL1 microduplication could be more frequent in 22q11.2DS patients.

To evaluate whether *KANSL1* duplication could be more frequent in 22q11.2DS patients with respect to control samples, CNV enrichment analysis was performed in the 22q11.2DS cohort and compared with respect to two independent cohorts of subjects without 22q11.2DS.

The second cohort corresponded to Chilean adult population controls subjects^19^ and showed that *KANSL1* duplication was present in 12.68% of samples, similar to the frequency in the 22q11.2DS cohort. A third cohort corresponding to 40 individuals with congenital anomalies and/or cognitive disabilities who had chromosome microarray (CMA) for clinical reasons^20^ was also studied. Eight of these 40 patients had the *KANSL1* duplication (20%) and five (12.5%) had CHD. Only one patient had both traits, the duplication of the *KANSL1* gene and CHD (p value = 1, OR = 0.85, CI = 0.015–10.37). These results indicate that *KANSL1* duplication and CHD are not statistically associated in patients without 22q11.2DS.

### CNV validation by MLPA

To validate the microduplication in *KANSL1* by an independent technique, MLPA analysis in all samples with 22q11.2DS using SALSA kit P443 (MRC Holland) was performed. This kit contains probes for all 15 exons of the *KANSL1* gene. The analysis of the samples showed that the duplication includes at least exons 1, 2 and 3 of the *KANSL1* gene. Nevertheless, the design of MLPA probes does not allow to define the exact breakpoints of the duplication.

### Biological interactions networks

If *KANSL1* is a modifier of CHD in 22q11.2DS patients, there must be a biological relationship with genes located in the 22q11.2 deletion region. To examine this hypothesis, a series of bioinformatic tools related to biological networks were used. Importantly, to be more stringent, only data with experimentally-validated biological interactions were considered.

**Figure 3:**
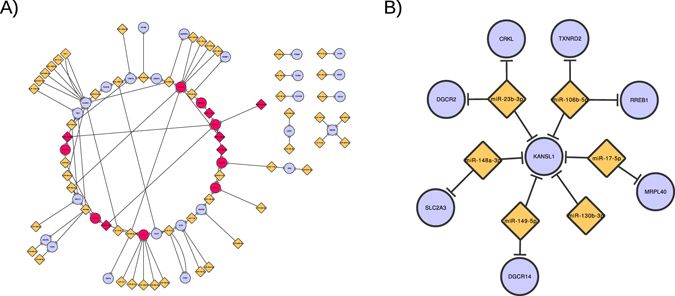
miRNA-mRNA interactions networks. The yellow diamonds represent miRNAs and the light purple, mRNA targets. The edge connecting two nodes indicate regulation. (**A**) The red filled nodes correspond to miRNAs, *KANSL1* and genes of deletion co-regulated by the same miRNAs in the context of the whole network. (**B**) Sub-network showing only *KANSL1* interacting module. The graphs were generated in Cytoscape 3.2 and drawing using the Circle Layout.

As a first approach, an analysis was conducted to determine existing protein-protein interaction between KANSL1 and protein-coding genes located in 22q11.2 deletion using GeneMANIA^21^. This tool showed no direct interactions between KANSL1 and protein-coding genes located in 22q11.2 deletion. Therefore, a second approach included the search for regulatory interactions, and a miRNA-mRNA network was constructed. *KANSL1* and the 46 protein coding genes in the 22q11.2 deletion region were used as input in miRWalk database to identify miRNAs that regulate these genes^22^. A miRNA-mRNA network was constructed using mRNA as the target node and the corresponding miRNA/s pairs found in the miRWalk database as the source node. This analysis showed that *KANSL1* is regulated by miR-106b-5p, miR-148a-3p, miR-23b-3p, miR-17-5p, miR-149-5p and miR-130b-3p. These miRNAs also regulate *DGCR14, DGCR2, TXNRD2, MRPL40* and *CRKL* genes, which are included in the 22q11.2 deletion. Moreover, in a second miRNA-mRNA network analysis, previously reported modifier genes (*MINA*, *RREB1*, *KDM7A* and *JMJDC1* and *SLC2A3*)^10, 12^ were also included. Interestingly, *SLC2A3* and *RREB1* genes, which are not included in 22q11.2 deletion, are regulated by the same miRNAs that regulate *KANSL1* (Fig. 3). To assess whether these results could occur by chance, the analyses was repeated using 50 random genes in network constructions. No shared miRNA regulation between random genes and those in the deletion region was observed.

## Discussion

We report the results of a genome-wide analysis in search for cardiac modifier CNVs in regions outside the 22q11.2 deletion using high-density arrays in a case-control study design. Results showed that a microduplication that contains at least the first 3 exons of the *KANSL1* gene is statistically associated with the presence of CHD in 22q11.2DS patients and biologically related with modifier genes and genes located in 22q11.2 deletion region. Even though statistical association did not reach the multiple hypothesis testing correction, the integration of the top ranked p-values with interaction data gives a robust combination of the results in a biological context. Moreover, other studies have shown that even when a non-statistically significant p-value is obtained, the use of biological networks gives important information related to the condition^12, 23–25^.

Findings show that *KANSL1* duplication is a common CNV, seen in approximately 12% of a large group of Chilean individuals, indicative that by itself it is frequent and benign, but that in combination with 22q11.2 deletion, it increases the risk of CHD.

KANSL1 is a protein-coding gene that belongs to a histone acetyltransferase (HAT) complex. This gene is encoded by KAT8, modifies the acetylation state of histone H4, mainly H4K16, and plays a role in the control of gene expression^26, 27^. Haploinsufficiency of this gene is implicated in Koolen-De Vries syndrome (KDVS, OMIM #610443)^28^. Interestingly, 39% of KDVS patients have CHD, mainly atrial septal defects or ventricular septal defects^29^. Analysis of the 17q21.31 region and characterization of KDVS patients without deletion of 17q21.31 show that SNVs located in *KANSL1* genes can be the cause of this syndrome^28^. Moreover, one of every four patients with point mutations in *KANSL1* has CHD, suggesting that this is a CHD-related gene, although further functional studies are needed to confirm this link^28^.

CNVs in *KANSL1* may be associated with CHD, with deletions causing KDVS and duplications acting as modifiers of the cardiovascular phenotype in 22q11.2DS. The literature reports show the effect of duplications in *KANSL1* gene in individuals with development delay, microcephaly and mild dysmorphic features^30–32^. These duplications span at least the complete *KANSL1* sequence and the microtubule-associated protein Tau gene (*MAPT*), which differs from the partial duplications found in 22q11.2 patients in this research. Mutations in *MAPT* gene are described in neurodegenerative disorders such as Alzheimer’s disease; therefore, it is highly likely that the duplicated region of *MAPT* could be involved in the phenotype of 17q31.21 duplication patients. However, patients with 17q21.31 microduplication present milder phenotypes than patients with the microdeletion, suggesting that microduplications cases are probably unascertained^30, 31^.

In the group of 40 patients with CMA, the duplication was frequent but not associated with CHD. However, in the group of patients with 22q11.2DS, the duplication was associated with CHD. This suggests that *KANSL1* microduplication, in combination with 22q11.2 deletion, increases the risk of developing CHD. The effects of the partial duplication in gene function and mechanisms of the interaction are to be elucidated; however, it is known that *KANSL1* is an important factor involved in the epigenetic modifications of histones. Variants in other genes with similar function affect conotruncal heart phenotypes in patients with 22q11.2DS^13^.

The relationship between histone modifier genes and heart disease has been previously described. It is widely known that epigenetic regulation of gene expression is mainly controlled by dynamic modification of chromatin structure^33^. This regulation mechanism is important during heart development as has been demonstrated in model organisms^33, 34^. Genes that encode proteins involved in binding or modification of histones proteins have been implicated as disease genes causing heart defects^35^. These data support the idea of a link at a functional level between normal heart development, heart defects, and epigenetic regulation. Moreover, Kabuki (OMIM #147920) and CHARGE syndrome (OMIM #214800) are two examples of diseases that have CHD among other traits in which responsible genes are chromatin modifiers^36, 37^.

In addition, the miRNA-mRNA network analysis revealed a common regulation between some genes in the 22q11.2 deletion region and *KANSL1*. Interestingly, other genes previously reported as modifiers of the CHD phenotype are regulated in the same module, showing a biological link between modifiers and genes located in 22q11.2 deletion region. This suggests that genes belonging to a common miRNA regulatory network could act as genetic modifiers, and variation in any one of these genes increases the risk of CHD. In this context, miRNA regulatory networks are an emergent field in 22q11.2DS research. The *DGCR8* gene, located in the 22q11.2 deletion, is involved in miRNA biogenesis since it is the cofactor for nuclease DROSHA, which processes primi-RNAs fold into hairpin structures containing imperfectly base-paired stems. DROSHA is an important regulator of miRNA biogenesis, suggesting that miRNA networks could be deregulated during development. Some miRNAs, including miR-150, miR-194 and miR-185, have been reported as differentially expressed in individuals with 22q11.2DS compared to controls^38^. It was proposed that this is related to *DGCR8* haploinsufficiency. Importantly, the expression level of some miRNAs correlated with a set of phenotypes, including heart defects, brain measures, and thyroid abnormalities, suggesting that the deregulated miRNAs may contribute to these characteristics^38^. Results in this study suggest that specific miRNAs regulate genes within the deletion as well as modifiers elsewhere in the genome, increasing the risk of CHD.

In summary, this study presents robust data showing that a duplication in the 17q31.21 region spanning the exons 1–3 of *KANSL1* gene is statistically associated with CHD in Chilean patients with 22q11.2DS. Moreover, *KANSL1* and genes located in 22q11.2 deletion share regulatory mechanisms mediated by miRNA. Future functional studies are needed in order to identify the exact mechanism of CHD mediated by *KANSL1*.

## Materials and Methods

The study and subject recruitment process were approved by the Ethics Committee of all the Institutional Review Boards at participating institutions (Facultad de Medicina, Clínica Alemana at the Universidad del Desarrollo, Hospital Padre Hurtado, Hospital Dr. Luis Calvo Mackenna, Hospital Dr. Roberto del Río, and Fundación Gantz). The study was carried out in accordance with the principles of the Declaration of Helsinki. Written informed consent was obtained from all patients and/or parents.

### Subject Cohorts

Blood samples were obtained from 253 patients diagnosed with 22q11.2 microdeletion demonstrated previously by fluorescence *in situ* hybridization (FISH) in clinical cytogenetic laboratories (Cohort 1). Clinical information including echocardiogram reports was collected from medical records.

The CEL files of cohorts 2 and 3 were obtained from previous studies: cohort 2 corresponds to Chilean population controls without 22q11.2DS genotyped with Affymetrix SNP 6.0 arrays^19^ and cohort 3 corresponds to Chilean patients without 22q11.2DS who had CMA with Affymetrix CytoScanHD arrays due to congenital anomalies or development disabilities^20^ according to ASHG guidelines^39^.

### Genotyping with Affymetrix SNP 6.0 array

Genomic DNA was purified from whole blood samples using AxyPrep blood genomic DNA kit (Axygen, Corning, NY, USA). Processing of genomic DNA and array hybridization was performed in accordance with manufacturer’s recommendations. Affymetrix Human SNP 6.0 array (Affymetrix, Santa Clara, CA, USA) contains 1.8 million genetic markers and more than 946.000 non-polymorphic CNV probes. Fluorescence intensities were quantified by Affymetrix Array Scanner 3000 7 G. Affymetrix Gene Chip Console (AGCC) software was used for data management and quality controls checks.

### CNV calling and filters

Every sample that passed standard SNP QC procedures according to Affymetrix Genotyping Console was entered into the CNV analysis pipeline. These samples were divided into two groups according to cardiac phenotype: patients with CHD (CHD, n = 133) and patients with normal cardiac anatomy (No CHD, n = 120). Affymetrix Power Tools software was used to compute LRR and B allele frequency measures. CNV calls were done with PennCNV software standard procedure^14^, using the standard hg18 “all” PennCNV hidden Markov model, population frequency of B allele files and GC model correction for the Affymetrix SNP 6.0 arrays available from PennCNV web-page.

Samples that failed PennCNV quality control were excluded, beginning with samples with LRR standard deviation >0.45. Samples that failed QC after the wave adjustment procedure were also removed. PennCNV frequently generates a great number of CNV calls for samples with very low quality even if LRR standard deviation appears normal, so the samples that generated over 100 CNV calls were also removed. Given that some centromeric and telomeric regions are not well mapped, potentially resulting in CNV-calling errors in these regions, they were removed from the analysis by delimiting them to a 500 kb window. Finally, genomic regions encoding immunoglobulin genes have previously shown be potential sites of false-positive PennCNV calls and were removed using genomics coordinates as suggested in the PennCNV software website^14^.

### Hierarchical sorting of CNV with bioinformatics approach

In order to identify gene or genes located in CNV regions that could potentially act as genetic modifiers of heart anatomy, the PhenogramViz plugin^17^ of CytoScape^18^ was used. This plugin facilitates a phenotype guided interpretation of CNVs by using the integrated cross-species phenotype ontology Uberpheno. The results were visualized as gene-to-phenotype association in a network of nodes and edges called phenograms. The UCSC Genome Browser LiftOver tool was used to convert CNV coordinates to the GRCh37/hg19 build^40^. A phenogram was created using the coordinates of CNVs of lowest p-value (p < 0.05) obtained with ParseCNV^15^ and the HPO terms “Tetralogy of Fallot” (HPO:0001636) and “Interrupted aortic arch” (HPO:0011611). The resulting phenogram was visualized in CytoScape 3.2.1.

### CNV validation of *KANSL1* microduplication by MLPA

Validation analysis was performed in samples from patients with 22q11.2DS using the SALSA MLPA kit P443 (MRC-Holland, Amsterdam, Netherlands), ABI-Prism 310 (Applied Biosystems, Foster City, CA) for capillary electrophoresis, and GeneMaker 2.0 software (SoftGenetics, State College, PA) to ascertain CNVs. Data was population-normalized, and probe ratios below 0.75 were considered as the threshold for deletion, while probe ratios above 1.25 were regarded as an indication of duplication. Validation was performed in 253 samples, with 7 showing results in cutoff limits; these were excluded from subsequent MLPA statistical analysis.

### Assessment of *KANSL1* microduplication frequency in non-22q11.2DS controls

To determine the frequency of *KANSL1* microduplication in patients without 22q11.2DS, a second set of data from Affymetrix SNP 6.0 microarray originating from population controls (cohort two) was employed^19^. The cardiac phenotype information in these individuals is unavailable. The CNV filter and calling pipeline for this cohort of patients were the same as for 22q11.2DS cohort. The CNVs were identified, and the frequency of *KANSL1* microduplication was calculated. In addition, data from a third cohort^20^ of patients with available cardiac phenotype information was processed using ChAS 2.0 software (Affymetrix, Santa Clara, CA). The association between CHD phenotype and the presence of *KANSL1* microduplication was calculated by Fisher’s exact test in the corresponding cohorts.

### Biological interaction of *KANSL1* and genes located in 22q11.2 deletion

To identify a link between *KANSL1* gene and genes located in the deleted region in 22q11.2DS, integrative bioinformatics approaches were employed. First, protein-protein interactions between KANSL1 and proteins coded by genes located in the deletion region (3 Mb) were analyzed using the GeneMANIA tool, and only validated protein-protein interactions were used as edge connections^21^. A second approach assessed whether *KANSL1* and genes located in 22q11.2 deletion could be involved in common regulatory mechanisms, using miRWalk database^22^, which has information about miRNA-mRNA pairs that have been experimentally validated. Briefly, a search for all mRNA-miRNA pairs for all genes located in 22q11.2 deletion and *KANSL1* was done in the miRWalk webpage (http://zmf.umm.uni-heidelberg.de/apps/zmf/mirwalk2/). Genes previously described as modifiers, including *SLC2A3*, *MINA*, *RREB1* and *JMJDC1* were used in this analysis^11, 13^. To avoid finding regulatory interactions due to chance, the same procedure was repeated using 50 random genes and genes located in 22q11.2 deletion. The list of random genes was extracted from the HUGO database using an in-house script. These lists of miRNA-mRNA pairs were used to construct miRNA-target regulatory networks, which were visualized and analyzed with Cytoscape 3.2 as published^41^.

### Statistical Analysis

A case/control association analysis was performed using ParseCNV, in which the main parameters were set by default using 1000 permutations. For CNV burden analysis, the number and size of deletions and duplications in CHD and no-CHD samples were counted. A Shapiro-Wilk test for normality of the data was performed, followed by a Wilcoxon rank sum test to compare CNV burden data between groups. The OR and CI of CNVs were calculated using the *epitools* package in R. All these analyses were done in R 3.2. software for statistical computing (Vienna, Austria).
